# Trends in social mobility depend on the competition between status and money

**DOI:** 10.1073/pnas.2200687119

**Published:** 2022-03-09

**Authors:** David B. Grusky

**Affiliations:** ^a^Center on Poverty and Inequality, Stanford University, Stanford, CA 94305

In all present-day societies, newborns would do well to care about where the stork drops them, as it profoundly affects their life chances. If the stork delivers a child to parents who work in professional, managerial, or other high-status occupations, the child can expect to be lavished with all manner of advantages (e.g., elite education), with these advantages then increasing their chances of ending up in a high-status occupation themselves. This type of birth lottery is inconsistent with a commitment to run a fair competition in which all children, no matter the circumstances of their parents, have the same chance of getting ahead.

Although there is a massive literature documenting the effects of the birth lottery, we know rather less about how that lottery is changing as our institutions adapt to the late-industrial world. In the PNAS article “Trends in Social Mobility in Postrevolution China,” Xie et al. (1) provide an especially valuable window into such long-term trends within a country, China, that makes up nearly one-fifth of the world’s population.

This paper contributes to our knowledge about long-term trends in two important ways. The first conclusion of Xie et al. (1) is that social mobility in China increased due to the “rapid industrialization” that shifted the labor force out of the low-mobility farming sector during the last 40 y. The second conclusion from their paper is that lurking behind this result is another force—that of marketization—that works in the opposing direction. When the effects of industrialization are netted out (by excluding workers born into the farm sector), Xie et al. (1) show that the trend in mobility shifts direction to reveal a decline. In recent years, many scholars have fretted that mobility in the United States may be declining (2), whereas Xie et al. (1) show that, if you want to find decline, you had best look to China rather than the United States.

These two key results suggest a simple framework for understanding the moving forces behind long-term trends. Although there are many sociological theories of mobility, they are typically tailored for specific countries or time periods and are accordingly cast in idiosyncratic terms that disguise the same moving parts lying behind all of them. The field has featured, for example, market transition theories to explain how the shift out of socialism affected mobility (3), theories of industrialism to explain how economic development affected mobility (4), and theories of marketization to explain how the spread of neoliberal institutions affected mobility (5). These theories, all of which play a part in Xie et al.’s (1) account, can be unified by refocusing on the meso-level elements that each of them invokes (see, e.g., ref. 6 for related mathematical models).

As shown in Table 1, there are four such meso-level elements (social status, family business status, economic resources, and sociocultural resources), each of which is distributed and institutionalized in ways that affect the amount of social mobility. The usual macro-level theories (marketization, industrialization, and market transition) invoke these elements in the various ways referenced below. The main point of Table 1, drawing on the insights of Xie et al. (1), is that the future of social mobility depends on a contest between 1) inequalities organized around status and the rules governing how status affects mobility and 2) inequalities organized around the distribution of economic resources and the rules governing how economic resources affect mobility. The former status-based forces are becoming less prominent (albeit very fitfully and imperfectly) and thus operate to increase social mobility, while the latter economic-based forces are becoming more prominent (again with much cross-society variability) and thus operate to reduce social mobility. The future of mobility hinges in this sense on the outcome of a competition between status-based and resource-based inequalities. In the following four sections of this commentary, these meso-level elements (two of which are status-based and two of which are resource-based) are reviewed in turn, with a focus on their likely contribution to the overall trend in social mobility over the next century or longer.

**Table 1.** The competition between status and resources in determining long-term trends in social mobility*

Status or resource	Compositional changes		Changes in effects	
	Illustrative compositional mechanism	Implication for rank–rank correlation	Illustrative change in effect	Implication for rank–rank correlation
Status-based organization				
Social status (e.g., race, ethnicity, gender, immigrant status, party status, spatial status)	Decline in size of rural population (SS1)	Reduction	Weakening of *hokou*; decline in racial or ethnic discrimination (SS2)	Reduction
Family business status^†^(e.g., farming business)	Decline in size of farming sector (FS1)	Reduction	Integration of farm and nonfarm sectors via, e.g., improved transportation systems (FS2)	Reduction
Resource-based organization				
Economic resources (e.g., income, wealth)	Growing income or wealth inequality ... such that top occupation ranks have relatively more money (ER1)	Increase	Marketization of opportunity makes parental economic resources more valuable (ER2)	Increase
Sociocultural resources (e.g., information, lifestyles, networks)	Growing sociocultural inequality ... such that top occupation ranks have relatively more sociocultural resources (SR1)	Increase	Growing sociocultural preconditions for access to opportunity (SR2)	Increase

^*^Following Xie et al. (1), social mobility is assumed to be measured with a rank–rank correlation, although the mechanisms laid out here are relevant for many other measures of relative mobility.

^†^The intergenerational transmission of a business should be distinguished from the intergenerational transmission of liquid wealth. The former is an example of status-based rules governing access to occupational roles (i.e., “status-based mobility”), while the latter pertains to the resources that can be bundled with a particular occupation (i.e., “resource-based mobility”).

## Status-Based Inequalities

The first meso-level element in Table 1 refers to the effects of organizing inequality around the various social statuses (e.g., race, ethnicity, and spatial status) that frequently appear in contemporary society. Following Max Weber (7), status groups can be understood as socially constructed categories that 1) have well-defined rules governing who can enter or exit the categories (i.e., rules of social closure), 2) entail different lifestyles by virtue of such closure, 3) are differentially ranked and honored, and 4) are subject to institutionalized practices (e.g., laws and norms) that affect whether members can gain access to particular roles or occupations. For our purposes, it is this last feature of status groups—their effects on access to roles or occupations—that is all-important. It is precisely these access-governing laws, norms, and rules (e.g., Jim Crow laws, status-based hiring discrimination, and *hokou*) that inhibit intergenerational mobility by preventing members of status groups from exiting the occupations to which they are historically consigned.

Although status groups can be constructed around physical traits (i.e., racial groups), national origins and traditions (i.e., ethnic groups and immigrant groups), or political party membership, Xie et al. (1) emphasize the especially important role, within China, of spatially defined categories built around the distinction between rural and urban residents. The *hukou* system regulates where people live by separating them into rural and urban “castes” and thereby controls access to the most remunerative occupations (which are disproportionately in urban areas). Because *hokou* status is inherited by children, it is a potentially strong status-based mechanism for reducing intergenerational mobility (as children born into a rural *hokou* status cannot as readily secure desirable urban occupations).

As indicated in Table 1, there are two ways in which *hokou*’s mobility-reducing effect can be undermined, the first being the simple “compositional effect” of a secular decline in the size of the rural population (see the cell labeled SS1). If ever-fewer people are limited by the mobility-reducing effect of a rural *hokou* status, then of course its overall role in reducing social mobility becomes more circumscribed. The second main way that *hokou* can be undermined is to reduce its regulative power on opportunities to secure remunerative occupations (see the cell labeled SS2). The limiting case here would of course be the outright elimination of the household registration system itself. Although the *hokou* system remains intact to this day, Xie et al. (1) describe various ways in which its power over occupational opportunities has been weakened.

The more general point is that status-based inequalities are increasingly viewed as arbitrary, unfair, and illegitimate (because they are not merit-based) and are therefore facing strong challenges in many contemporary societies. These challenges, although ongoing, of course coexist with powerful efforts to retain and elaborate status-based organization (e.g., populism). If the latter efforts win out and bring about a strengthening (rather than weakening) of status-based constraints on accessing occupations, then the future of social mobility is exceedingly bleak because in this case all forces in Table 1—not just resource-based ones—are working in concert to reduce mobility.

## Family Business Inequalities

In the second row of Table 1, the “family business” status group is singled out, as it has played an especially critical role in the process of industrialization. The most common family business, at least until recently, has been a family farm that is passed on from one generation to the next (via formal property ownership or through rules of social closure in which collectively owned or state-owned farms are nonetheless “passed on” intergenerationally). The family business is a potent force for immobility because it wraps the family around a particular business and thus very organically combines childhood socialization and training with occupational socialization and training. In societies with formal property ownership, these social processes are combined with legalized intergenerational transmission of a business, again with mobility-reducing effects.

The main force undermining this “family business” effect within China is, as Xie et al. (1) very elegantly lay out, a wholly compositional one in which industrialization works to reduce the size of the farming sector (see the cell FS1). This process increases mobility by reducing the proportion of children who grow up in family businesses that limit their aspirations and narrowly focus their training. Although Xie et al. (1) emphasize this compositional process, it could in principle be complemented by various institutional changes, such as the integration of farm and nonfarm economies (e.g., improved transportation and cross-sector networks), that additionally serve to reduce the “effect” of an origin farm status on the child’s opportunities (see the cell FS2).

## Economic Resource Inequalities

The foregoing status-based sources of immobility thus face fundamental challenges in contemporary systems that might lead one to hypothesize that social mobility should gradually increase. If these status-based sources were the only sources of immobility, this would indeed be a compelling hypothesis. But of course they are not the only sources.

The main confounding dynamic is that, just as such status-based sources of immobility start to weaken, they have often been replaced by rising economic inequalities that can reduce mobility. The twofold point here is that 1) between-occupation income inequality is increasing in many societies, and 2) this rising resource-based inequality has a retarding effect on mobility. As Mitnik et al. (2) discuss, the growth of income inequality in the United States means that parents in top-ranked occupations (e.g., managers and professionals) have relatively more resources to assist their children, with the result that their children are increasingly likely to end up in a top-ranked occupation themselves (see the cell ER1). This outcome is not an effect of marketization per se but of rising inequality (that marketization may or may not bring about).

The latter compositional process can, however, be complemented by bona fide marketization effects, assuming that marketization refers, in particular, to putting opportunity on the market (see the cell ER2). If a society’s institutions are recast, for example, to increasingly require money to access high-quality training, then there will be a growing reproductive payoff to the economic resources that parents in top-ranked occupations command. In Grusky et al. (5), this move toward “putting opportunity on the market” is described in detail, with a special focus on the growing cost of building a resume for one’s children that will appeal to selective tertiary institutions. This marketization of access to training and credentialling takes the form in China of costly after-school tutoring, test preparation services, and foreign boarding schools. These opportunity markets, which appear to be emerging in countries throughout the world, allow well-off parents to convert their money into high-quality resumes for their children and thus create the appearance that merit just happens to coincide with money. By contrast, status-based inequalities in access to training are more obviously inconsistent with a commitment to merit-based allocation, thus making them more vulnerable to delegitimation.

## Sociocultural Resources

The final row of Table 1 pertains to the distribution of sociocultural resources rather than economic resources. Because information, networks, and other sociocultural resources are unequally distributed across occupations, a key mobility-restricting dynamic is that of predicating access to desirable occupations on such resources. This predication could take the form, for example, of university admission committees or hiring managers selecting on the dominant-group accent, the dominant-group leisure tastes, or the dominant-group networks. Although it is well-established that economic resources (e.g., income) are increasingly concentrated in top-ranked occupations, there is less evidence of an analogous concentrating trend for sociocultural resources. There are nonetheless various worrying developments, such as the growth of income-based residential segregation, that could in principle parlay into growing sociocultural inequalities.

It is possible, therefore, that both types of inequality (i.e., economic and sociocultural) will grow swiftly enough to offset the mobility-increasing effects of a decline in status-based organization. In Xie et al. (1), the decline in status-based organization was clearly the overwhelming force, but there is no guarantee that it will continue to overcome the combined effects of 1) rising inequality and 2) ample opportunities for the well-off to buy opportunities for their children with the “extra money” that rising inequality affords them.

## Conclusions

It bears recalling Marx and Engel’s (8) famous passage: “The bourgeoisie, whenever it has got the upper hand, has put an end to all feudal, patriarchal, idyllic relations. It has pitilessly torn asunder the motley feudal ties that bound man to his ‘natural superiors’ and has left remaining no other nexus between man and man than … callous ‘cash payment.’” The formulation laid out in Table 1 captures this ongoing contest between status-based and resource-based organization and suggests that it drives the future trajectory of social mobility. The upshot, in other words, of Table 1 is that late-industrial mobility may be growing less responsive to an individual’s status (e.g., spatial, ethnic, racial, or immigrant status) but more responsive to their capacity to purchase the training needed for high-status occupations. Because that capacity is strongly associated with origin occupations, the latter dynamic can readily offset the former and, in principle, lead to a reduction in the amount of mobility. It is very difficult to assess which of these two forces will win out, whether the outcome of this competition will be the same in all societies, and whether an antineoliberal movement delegitimating the “purchase of opportunity” will ever become ascendent. The trajectory of late-industrial mobility regimes is in this sense very open.
